# Trends in the Management of Small Renal Masses: A Survey of Members of the Endourological Society

**DOI:** 10.15586/jkcvhl.2017.82

**Published:** 2017-07-20

**Authors:** Anand Mohapatra, Aaron M. Potretzke, John Weaver, Barrett G. Anderson, Joel Vetter, Robert S. Figenshau

**Affiliations:** 1 Department of Urology, UPMC, Pittsburgh, PA, USA; 2 Department of Urology, Mayo Clinic, Rochester, MN, USA; 3 Division of Urologic Surgery, Washington University School of Medicine, St. Louis, MO, USA

**Keywords:** active surveillance, laparoscopic ablation, minimally invasive nephrectomy, robotic nephrectomy, small renal masses

## Abstract

Treatment modalities for small renal masses (SRMs) include open or minimally invasive radical or partial nephrectomy, and laparoscopic or percutaneous ablations. Members of the Endourological Society were surveyed to evaluate how practitioner and clinical practice characteristics may be associated with the management of SRMs over time. The survey assessed characteristics of urologists (recency of residency and fellowship training, clinical practice type and location, and treatment modalities available) and their management of SRMs over the past year and over the course of the year 5 years prior. Of the 1495 surveys e-mailed, there were 129 respondents (8.6%). Comparing the past year to 5 years prior, there was increasing utilization of robotic partial nephrectomy (p < 0.001) and robotic radial nephrectomy (p = 0.031). In contrast, there was decreasing utilization of open partial nephrectomy (p < 0.001), open radical nephrectomy (p = 0.039), laparoscopic partial nephrectomy (p = 0.002), and laparoscopic radical nephrectomy (p = 0.041). Employment of laparoscopic ablation decreased (p = 0.001), but that of percutaneous ablation did not change significantly. For masses treated with image-guided therapy, there was increasing utilization of microwave ablation (p = 0.008) and decreasing usage of radiofrequency ablation (p = 0.002). Future studies should focus on the most effective treatment modalities based on provider, patient, and tumor characteristics.

## Introduction

With increased utilization of CT scans, ultrasound, and other diagnostic imaging techniques, the incidental detection of renal masses has become progressively more common (1–5). Commensurate with this trend, the incidence of small renal masses (SRMs) has steadily grown by as much as 3.7% annually since the 1970s (2, 6, 7). As evidenced by the American Urological Association (AUA) guidelines, numerous options exist for the management of SRMs. These include open or minimally invasive radical or partial nephrectomy, percutaneous cryo-, microwave, or radiofrequency ablation, and active surveillance (8). However, many of these treatment options involve newer technologies and have only recently become more widely utilized. For instance, in 1998, urologists performed a radical nephrectomy for 58% of cortical renal masses up to 2 cm and 76% between 2 and 4 cm. Over the next decade, these numbers gradually declined to 29% and 48%, respectively. Correspondingly, the employment of partial nephrectomy, ablation, and active surveillance has increased (9). Despite an increase in partial nephrectomy over time, recent literature suggests that partial nephrectomy is still underutilized (10–13). Although changes in management of SRMs have been previously described, factors which may relate to current practice patterns have not yet been illustrated. This study sought to describe the clinical, surgical, geographic and temporal factors that may relate to management strategies by members of the Endourological Society who practice in the United States.

## Materials and methods

### Survey administration and audience

An electronic survey was designed to assess the characteristics of urologists, their clinical practices, and their management of SRMs both in the past year and during the year 5 years prior. The survey can be viewed in Supplementary File 1. Institutional Review Board approval was obtained. The survey was administered through the Washington University School of Medicine Research Electronic Data Capture (REDCap) system. It was distributed to all members of the Endourological Society with a registered e-mail address and residence within the United States as listed on the Endourological Society website (www.endourology.org). A unique URL ensured that each receipient could only respond once. A follow-up e-mail was sent after 2 weeks to those who had not responded. Individuals who appeared to be industry representatives based on the domain of their e-mail addresses were excluded.

### Statistical analyses

McNemar’s chi-square test compared availability of each modality in the past year and 5 years prior. Chi-square test of independence and Fisher’s exact test compared utilization rates of each modality in the past year and 5 years prior for the entire data set and subgrouped by practice and practitioner characteristics. Statistical significance was determined to be p < 0.05. All statistical analyses were performed using R v3.3.1 (14).

## Results

### Supplementary data

Supplementary data of this article, as supplied by the authors, are available as pdf on the journal’s website. They can be accessed via the following link: http://jkcvhl.com/index.php/jkcvhl/rt/suppFiles/82/0.

### Respondent characteristics

Of the 1495 surveys e-mailed, 450 were undeliverable. There were 129 respondents in total (8.6%). Demographics regarding the respondents and their practices are detailed in Table 1. Most respondents had completed a fellowship (70%), the most popular of which was endourology (53/129, 41%). Approximately half of the fellowship-trained respondents (44/87, 50.6%) completed fellowship in the past 5 years. Regarding the number of clinic patients seen per week, the most common response was 50–75 (44/129, 34.1%).

**Table 1:** Summary of respondent training and practice characteristics

Variable	Respondents (%) (N = 129)
Resident time frame	
0–5 years ago	56 (43.4)
6–10 years ago	28 (21.7)
11–15 years ago	20 (15.5)
>15 years ago	25 (19.4)
Fellowship training	
Endourology	53 (41.1)
Robotics	5 (3.9)
Reconstructive	4 (3.1)
Oncology	22 (17.1)
Transplant	1 (0.8)
Female urology	2 (1.6)
Infertility	0 (0.0)
Pediatrics	4 (3.1)
Research	5 (3.9)
Other	1 (0.8)
No fellowship	39 (30.2)
Fellowship time frame	
0–5 years ago	44 (50.6)
6–10 years ago	16 (18.4)
11–15 years ago	14 (16.1)
>15 years ago	13 (14.9)
Region	
West	24 (18.6)
Midwest	34 (26.4)
Northeast/New England	36 (27.9)
South	33 (25.6)
Other	2 (1.6)
Practice type	
Academic	63 (49.2)
Nonacademic, hospital based	23 (18.0)
Private practice	42 (32.8)
Practice setting	
Metropolitan	103 (80.5)
Nonmetropolitan	25 (19.5)
Number of patients per week	
<50	27 (20.9)
50–75	44 (34.1)
76–100	36 (27.9)
101–125	16 (12.4)
126–150	5 (3.9)
>150	1 (0.8)
Percentage of patients with BMI > 30	
<10	3 (2.3)
10–25	17 (13.3)
25–50	67 (52.3)
50–75	39 (29.7)
75–100	3 (2.3)

BMI, body mass index.

### Treatment modalities for SRMs

Data regarding treatment modalities employed by the entire cohort are available in Table 2. All treatment modalities except robotic surgery had similar availability to urologists in the past year compared to 5 years prior. Robotic surgery was more commonly available in the last year (96% vs. 83.5%, p = 0.002). Table 3 details the rates of usage for various SRM treatment modalities. Comparing 5 years prior with the past year, there was increasing utilization of robotic partial nephrectomy (p < 0.001) and robotic radical nephrectomy (p = 0.031). In contrast, there was decreasing utilization of open partial nephrectomy (p < 0.001), open radical nephrectomy (p = 0.039), laparoscopic partial nephrectomy (p = 0.002), laparoscopic radical nephrectomy (p = 0.041), and laparoscopic ablation (p = 0.001). Of the renal masses treated with image-guided therapy, the survey data showed decreased usage of radiofrequency ablation (p = 0.002) and increased usage of microwave ablation (p = 0.008) in the past year compared to 5 years prior. Table 4 provides an abridged summary of respondent practice patterns during the two time periods.

**Table 2:** Availability of treatment modalities for SRMs within the past year and 5 years ago

Available modality	Within the past yearRespondents (%) (N = 124)	Five years agoRespondents (%) (N = 85)	p
Robotic surgery	119 (96.0)	71 (83.5)	**0.002**
Laparoscopic surgery	119 (96.0)	82 (96.5)	0.852
Percutaneous ablation	113 (91.1)	75 (88.2)	0.494
Laparoscopic ablation	98 (79.0)	70 (82.4)	0.553
All available	92 (74.2)	56 (65.9)	0.194

**Table 3:** Usage rates of treatment modalities for SRMs within the past year and 5 years ago

Usage rate per modality	Within the past year Percentage of respondents (N = 124)	Five years ago Percentage of respondents (N = 85)	p
**Survey question: Of the SRMs that were treated, what percentage of cases were treated by…**			
Open partial nephrectomy			**<0.001**
0%	40.3	22.1	
10%	50.6	41.6	
≥25%	9.1	36.4	
Open radical nephrectomy			**0.039**
0%	44.2	31.2	
10%	46.8	55.8	
≥25%	9.1	13.0	
Laparoscopic partial nephrectomy			**0.002**
0%	84.4	63.6	
10%	6.5	19.5	
≥25%	9.1	16.9	
Laparoscopic radical nephrectomy			**0.041**
0%	26.0	18.2	
10%	32.5	23.4	
≥25%	41.6	58.4	
Robotic partial nephrectomy			**<0.001**
≤10%	10.4	36.4	
25%	23.4	27.3	
50%	37.7	23.4	
≥75%	28.6	13.0	
Robotic radical nephrectomy			**0.031**
0%	61.0	72.7	
10%	19.5	16.9	
≥25%	19.5	10.4	
Percutaneous ablation			0.865
0%	31.2	32.5	
10%	50.6	53.2	
≥25%	18.2	14.3	
Laparoscopic ablation			**0.001**
0%	81.8	63.4	
≥10%	18.2	36.4	
**Survey question: Of the SRMs that were treated with image-guided ablation, what percentage of cases were treated by…**			
Cryoablation			0.362
0%–10%	15.7	19.6	
25%–75%	29.4	23.5	
90%–100%	54.9	56.9	
Radiofrequency ablation			**0.002**
0%	60.8	45.1	
10%–50%	25.5	39.2	
75%–100%	13.7	15.7	
Microwave ablation			**0.008**
0%	76.5	94.1	
≥10%	23.5	5.9	

**Table 4:** A bridged summary of respondent practice patterns for SRMs within the past 5 years

Variable	Within the past year Respondents (%) (N = 124)	Five years ago Respondents (%) (N = 87)
Number of renal masses		
<5	6 (4.8)	4 (4.6)
5–10	10 (8.1)	6 (6.9)
11–20	20 (16.1)	11 (12.6)
21–30	15 (12.1)	17 (19.5)
31–40	22 (17.7)	14 (16.1)
>40	51 (41.1)	35 (40.2)
Unavailable treatment options		
Robotic surgery	5 (4.0)	14 (16.5)
Laparoscopic surgery	5 (4.0)	3 (3.5)
Percutaneous ablation	11 (8.9)	10 (11.8)
Laparoscopic ablation	26 (21.0)	15 (17.6)
All available	92 (74.2)	56 (65.9)
Active surveillance, percent		
0	3 (2.4)	6 (7.2)
10	65 (52.4)	51 (61.4)
25	43 (34.7)	23 (27.7)
≥50	13 (10.5)	3 (3.6)
Open partial nephrectomy, percent		
0	49 (42.2)	17 (21.3)
10	55 (47.4)	34 (42.5)
25	7 (6.0)	22 (27.5)
≥50	5 (4.3)	7 (8.8)
Open radical nephrectomy, percent		
0	59 (50.9)	24 (30.0)
10	46 (39.7)	46 (57.5)
25	10 (8.6)	8 (10.0)
≥50	1 (0.9)	2 (2.5)
Laparoscopic partial nephrectomy, percent		
0	96 (82.8)	51 (63.8)
10	8 (6.9)	16 (20.0)
25	7 (6.0)	8 (10.0)
≥50	5 (4.3)	5 (6.3)
Laparoscopic radical nephrectomy, percent		
0	30 (25.9)	14 (17.5)
10	40 (34.5)	18 (22.5)
25	38 (32.8)	36 (45.0)
≥50	8 (6.9)	12 (15.0)
Robotic partial nephrectomy, percent		
0	10 (8.6)	20 (25.0)
10	5 (4.3)	8 (10.0)
25	25 (21.6)	22 (27.5)
≥50	76 (65.5)	30 (37.5)
Robotic radical nephrectomy, percent		
0	72 (62.1)	58 (72.5)
10	23 (19.8)	14 (17.5)
25	15 (12.9)	6 (7.5)
≥50	6 (5.2)	2 (2.5)
Percutaneous ablation, percent		
0	37 (31.9)	27 (33.8)
10	61 (52.6)	42 (52.5)
25	18 (15.5)	10 (12.5)
≥50	0 (0.0)	1 (1.3)
Laparoscopic ablation, percent		
0	100 (86.2)	50 (62.5)
10	14 (12.1)	23 (28.8)
25	2 (1.7)	6 (7.5)
≥50	0 (0.0)	1 (1.3)

#### By practice type

Subgrouping the data by practice type (academic, nonacademic hospital-based, and private practice), there were no significant differences in the employment of the various treatment modalities in the past year. Five years ago, the usage of robotic radical nephrectomy differed among the practice types (p = 0.043); academic, private practice, and nonacademic hospital-based urologists reported utilization rates of 11.6%, 15%, and 0%, respectively, for ≥25% of SRM cases. There were no significant differences when comparing changes in utilization over time for the different practice types (Supplementary Table 1).

#### By practice setting

There were no significant differences in the utilization of treatment modalities based on practice setting (metropolitan and nonmetropolitan) in either time period. Of the respondents who utilized ablation, nonmetropolitan urologists increased usage of radiofrequency ablation (p = 0.04) and decreased usage of cryoablation (p = 0.05) at greater rates over the past 5 years compared to metropolitan urologists (Supplementary Table 2).

#### By fellowship training

Completed data and statistical analyses pertaining to treatment modality in relation to fellowship training versus no fellowship training can be found in Supplementary Table 3. Data separated by fellowship type are included in Supplementary Table 4a and 4b. Respondents who had completed a fellowship more often performed open partial nephrectomy in the last year than those without fellowship training (p = 0.029). Further, urologists who completed an endourology fellowship or no fellowship performed open partial nephrectomy and open radical nephrectomy less than urologists who completed an oncology or other fellowships (p = 0.001 and 0.025, respectively). Members of the Endourological Society who completed an endourology fellowships trended toward greater utilization of robotic partial nephrectomy in the past year when compared to urologists with other training backgrounds (when comparing utilization with greater than 75% of SRMs to utilization with less than 75% of SRMs), although this relationship did not reach statistical significance (p = 0.064). Of urologists who utilized image-guided therapy, fellowship-trained urologists were less likely to use cryoablation and more likely to use microwave ablation compared to non-fellowship-trained urologists in the past year (p = 0.028 and 0.018, respectively). In addition, of those who performed ablation procedures, 35.7% of endourology-trained urologists increased their implementation of microwave ablation over the past 5 years compared to 0% for other fellowship-trained and non-fellowship-trained urologists (p = 0.001).

#### By residency time frame

Urologists who completed residency at least 15 years ago had greater utilization of laparoscopic partial nephrectomy over the last year compared to those who completed residency more recently (p = 0.002). Otherwise, the data yielded similar utilization of the different treatment modalities both in the past year and 5 years prior (Supplementary Table 5a and 5b).

## Discussion

While open radical nephrectomy was formerly the standard of care (15), there now exist several effective options for managing SRMs. These include open or minimally invasive radical or partial nephrectomy, percutaneous cryo-, microwave, or radiofrequency ablation, and active surveillance (8). Many of these treatment modalities have only recently gained popularity, as they involve newer technologies and practices. This study explored the differences in utilization of these modalities in the past year and 5 years prior among members of the Endourological Society within the United States. Survey data demonstrated increasing usage of robotic partial and radical nephrectomy (p < 0.001 and p = 0.031, respectively) and microwave ablation (p = 0.008), and decreasing usage of other surgical treatments (p values < 0.05), radiofrequency ablation (p = 0.002), and laparoscopic ablation (p = 0.001).

Urologists trended toward increasing utilization of robotic partial and radical nephrectomy over the past 5 years (p < 0.001 and p = 0.031, respectively). In contrast, there was decreasing utilization of open partial nephrectomy (p < 0.001), open radical nephrectomy (p = 0.039), laparoscopic partial nephrectomy (p = 0.002), and laparoscopic radical nephrectomy (p = 0.041). These changes reflect the growing usage of robotic surgery in the management of SRMs (16, 17). Recent studies have suggested an underutilization of partial nephrectomy in general, but the availability of robotic technology facilitates an increase in the rate of partial nephrectomy (10–13). On a population level, Patel et al. showed that patients are more likely to undergo a partial nephrectomy if their urologist performs robotic surgery (17). There are well-described advantages of robotic partial nephrectomy compared with laparoscopic surgery; therefore, further displacement of laparoscopic partial nephrectomy would be appropriate. One large meta-analysis showed robotic partial nephrectomy to have shorter, warm ischemia time, shorter hospital stays, and lower rates of conversion to open surgery (18). Subjectively, robotic surgery allows for more precise surgical maneuvers and offers a relatively short learning curve compared with the laparoscopic approach (19, 20).

The diffusion of technology in urologic surgery has garnered debate in recent years. Although the adoption of new surgical techniques and platforms (i.e., robotic surgery) is paramount to continued innovation and improved patient outcomes, many have aptly argued that more fastidious research and evaluation of new technology is prudent. The widespread adoption of robotic partial nephrectomy, for example, has afforded a minimally invasive and nephron-sparing approach to many patients who may have otherwise undergone radical nephrectomy (12, 21). The potential benefits of partial nephrectomy and, specifically, minimally invasive partial nephrectomy are well established (22–28). In fact, it has recently been proposed that robotic partial nephrectomy is becoming the standard of care for SRMs. Previously, authors have argued that diffusion of minimally invasive and nephron-sparing surgery was, in fact, too hindered and dependent on surgeon preference, especially given its purported safety (16, 29, 30). Conversely, others have pronounced the process of robotic adoption as hurried and dubious, with little oversight or prospective randomized data to support its utilization. Comparative concerns may be drawn from the rapid dissemination of robotic prostatectomy. Parsons et al. demonstrated that during the early adoption of robotic prostatectomy, there was increased risk of patient safety indicators (adjusted odds ratio, 2.0; 95% CI, 1.1–3.7; p = 0.02) (31). Of course, large prospective randomized trials are costly, time-consuming, and onerous. Hence, the debate regarding the optimal evaluation and institution of new technology continues. From the present data, it certainly appears that the diffusion process has been significant over the last 5 years for both robotic partial nephrectomy (p < 0.001) and the newest form of percutaneous ablation—microwave (p = 0.008).

As urologic surgery continues to trend toward minimally invasive interventions, there has been heightened reliance on image guidance. Although open surgery remains a trusted and effective approach for the management of SRMs, the evolution of laparoscopic extirpation and ablation to robotic enucleation and percutaneous ablation has been demonstrative. Appropriately, the collaboration of urologists and radiologists is critical. The intraoperative interpretation of robotically controlled ultrasound is something with which many urologists have become comfortable. However, a cooperative partnership with radiologists affords the urologist the opportunity to have a “second opinion” intraoperatively for challenging cases. Furthermore, although percutaneous ablation, and particularly microwave ablation, has been championed by radiologists at many institutions, the urologist’s role remains vital in several ways. The identification of potential candidates for ablation is augmented by consultation with radiology. Moreland et al. described the experience of having both a urologist and a radiologist in the procedure suite for ablative cases (32). Furthermore, Welch et al. postulated that this relationship, commensurate with a higher volume of procedures, improves patient safety (33). The present survey illustrates that with this partnership and the appropriate diffusion of new research, radiofrequency utilization has decreased (Table 3, p = 0.02), while employment of cryoablation has remained steady (Table 3, p = 0.362), and usage of microwave ablation has increased (Table 3, p = 0.008). Emerging evidence conveys the inferiority of radiofrequency ablation relative to cryoablation (34, 35). Finally, the increased utilization of microwave ablation over the last 5 years and its postulated advantages (36) are likely the product of this productive, concerted relationship.

When comparing percutaneous ablation to laparoscopic ablation, survey data showed that usage of percutaneous ablation has remained relatively constant (p = 0.865), while the usage of laparoscopic ablation has decreased in recent years (p = 0.001). Theoretical advantages of the laparoscopic approach include placement of probes under direct visualization and treatment of anterior tumors. Benefits of the percutaneous approach include the avoidance of a general anesthetic, shorter recovery time, and the ability to perform the procedure on an outpatient basis. Given the similar oncologic and functional outcomes between laparoscopic and percutaneous ablation demonstrated by Zargar et al. (37), it is understandable that laparoscopic ablation has fallen out of favor over the past 5 years.

There are a few important limitations of this study. First, the low survey response rate may have biased results. Additionally, only surveying members of the Endourological Society likely resulted in a higher rate of respondents who were endourology trained. Because respondents were asked to remember their practice patterns retrospectively, recall bias may have also affected the data. Finally, although survey data allowed for associations to be made between practice patterns and provider characteristics, no conclusions could be drawn on why those associations exist.

## Conclusion

The availability and utilization of robotic surgery have increased over the last 5 years, whereas utilization of open and laparoscopic surgery has decreased. The employment of percutaneous ablation has remained the same, whereas that of laparoscopic ablation has decreased. Although all of these are acceptable treatment modalities for SRMs per the AUA guidelines, the authors hope that examining usage patterns and trends will spur discussion on the most effective treatment modalities based on provider, patient, and tumor characteristics.
